# Arbuscular mycorrhizal fungi reduce nitrous oxide emissions from N_2_O hotspots

**DOI:** 10.1111/nph.14931

**Published:** 2017-12-05

**Authors:** Kate Storer, Aisha Coggan, Phil Ineson, Angela Hodge

**Affiliations:** ^1^ Department of Biology University of York Wentworth Way York YO10 5DD UK; ^2^ Present address: ADAS High Mowthorpe Duggleby, Malton North Yorkshire YO17 8BP UK

**Keywords:** agriculture, arbuscular mycorrhizal fungi (AMF), greenhouse gas, hyphosphere, N cycle, nitrification, nitrogen (N), nitrous oxide (N_2_O)

## Abstract

- Nitrous oxide (N_2_O) is a potent, globally important, greenhouse gas, predominantly released from agricultural soils during nitrogen (N) cycling. Arbuscular mycorrhizal fungi (AMF) form a mutualistic symbiosis with two‐thirds of land plants, providing phosphorus and/or N in exchange for carbon. As AMF acquire N, it was hypothesized that AMF hyphae may reduce N_2_O production.
- AMF hyphae were either allowed (AMF) or prevented (nonAMF) access to a compartment containing an organic matter and soil patch in two independent microcosm experiments. Compartment and patch N_2_O production was measured both before and after addition of ammonium and nitrate.
- In both experiments, N_2_O production decreased when AMF hyphae were present before inorganic N addition. In the presence of AMF hyphae, N_2_O production remained low following ammonium application, but increased in the nonAMF controls. By contrast, negligible N_2_O was produced following nitrate application to either AMF treatment.
- Thus, the main N_2_O source in this system appeared to be via nitrification, and the production of N_2_O was reduced in the presence of AMF hyphae. It is hypothesized that AMF hyphae may be outcompeting slow‐growing nitrifiers for ammonium. This has significant global implications for our understanding of soil N cycling pathways and N_2_O production.

## Introduction

Agricultural soils are a major source of the globally important greenhouse gas, nitrous oxide (N_2_O), a gaseous product of the nitrogen (N) cycle (Singh *et al*., 2010; Hartmann *et al*., 2013). In fact, the marked global N_2_O atmospheric concentration increases between 1940 and 2005 were predominantly a result of increased use of N‐based fertilizers in agricultural systems (Park *et al*., 2012). N_2_O also has a long pertubation lifetime of 121 yr (Hartmann *et al*., 2013), and thus it is essential that we understand the soil‐derived fluxes of N_2_O, as, unlike shorter‐lived greenhouse gases (e.g. CH_4_; Hartmann *et al*., 2013), any changes in the atmospheric concentration of N_2_O will have long‐term effects. Consequently, N_2_O is viewed as an immediate target to achieve greenhouse gas reductions (Wuebbles & Hayhoe, 2002; Reay *et al*., 2012). However, in order to achieve such reductions, an enhanced understanding of the major sources and sinks of N_2_O is urgently required.

In recent years, our understanding of N_2_O production in soil systems has significantly improved, mostly as a result of the development of isotopic methods for tracing the sources of N_2_O (Baggs, 2008; Kool *et al*., 2011a; Ostrom & Ostrom, 2011). The rate of N_2_O production is predominantly controlled by the availability of the inorganic N source (Hino *et al*., 2010), O_2_ (Bollmann & Conrad, 1998), and other factors that influence microbial activity (e.g. temperature, carbon (C) availability and pH; Bollmann & Conrad, 1998; Prosser, 2007; Thomson *et al*., 2012). In addition, recent evidence has revealed that N_2_O reduction is not only confined to denitrifers. Other commonly occurring soil bacteria and archaea may also utilize exogenous N_2_O, including under aerobic conditions, even though they lack the preceding steps in the denitrification pathway (Sanford *et al*., 2012; Jones *et al*., 2014). Therefore, it follows that the net N_2_O emitted from soils will be influenced by the presence of microorganisms.

Arbuscular mycorrhizal fungi (AMF) are a key group of soil microorganisms that form symbiotic associations with the majority of land plants (Smith & Read, 2008). Moreover, it is now widely acknowledged that these fungi play a previously unrecognized role in nitrogen (N) cycling, and can both aquire N for their host plant (Barrett *et al*., 2011; Herman *et al*., 2012) and have a substantial N requirement themselves (Hodge & Fitter, 2010). There is also evidence of reduced nitrate (NO_3_^−^) leaching in the presence AMF (Asghari & Cavagnaro, 2012; Cavagnaro *et al*., 2015; Köhl & van der Heijden, 2016). Alongside NO_3_^−^, a major output of the N cycle is the potent greenhouse gas, N_2_O. Therefore, it might be expected that these fungi might influence the availability of N substrates (ammonium (NH_4_^+^) and NO_3_^−^) for N_2_O production. AMF have been shown to be able to acquire both NH_4_^+^ and NO_3_^−^, although it appears they may prefer the more energetically attractive NH_4_^+^ (Govindarajulu *et al*., 2005; Hodge & Storer, 2015). If these fungi compete effectively with other microorganisms for these inorganic N forms then this could reduce the availability of N substrates for N_2_O producers, leading to a reduction in N_2_O emissions. There is some circumstantial evidence to suggest this may be the case. For example, Bender *et al*. (2014) found a reduction in N_2_O fluxes from soils influenced by AMF‐colonized roots when compared with soils influenced by roots alone. N_2_O fluxes are also reduced when rice plants in draining paddies are arbuscular mycorrhizal (Zhang *et al*., 2015). Collectively, these studies suggest that AMF may alter N_2_O emissions in conventional agricultural soils but, thus far, it has not been determined if this is mediated through physiological changes in the AMF‐colonized roots, or as a direct result of the AMF themselves. If AMF hyphae can directly reduce N_2_O production, this could have significant implications for global N_2_O production and our understanding of soil N cycling.

Arbuscular mycorrhizal fungi hyphae have previously been demonstrated to proliferate in organic matter patches (e.g. Hodge *et al*., 2001; Barrett *et al*., 2014; Hodge, 2014) and have been shown to take up and transfer N in the inorganic form from these patches to their host plant (Leigh *et al*., 2009; Hodge & Fitter, 2010). The two studies described here followed a similar experimental design to that of Hodge & Fitter (2010) using dried, milled *Zea mays* L. leaves mixed with an agricultural soil (which had a high N_2_O production rate; Storer, 2013), to create organic matter ‘patches’. These organic matter patches represent ‘N_2_O hotspots’ which commonly occur in natural systems (Cowan *et al*., 2015). Both experiments tested the hypothesis that AMF hyphae would reduce N_2_O production from the organic matter patches, while the second experiment further examined the hypothesis that a reduction in N_2_O production was a consequence of reduced nitrification rates in the presence of AMF hyphae.

## Materials and Methods

### Microcosm design and growth media

To test the hypothesis that N_2_O production was reduced in the presence of AMF hyphae, two experiments were established under glasshouse conditions using compartmented microcosm units. Expt 1 was designed to determine the impact of AMF hyphae on N_2_O production, whereas Expt 2 was designed to determine whether AMF hyphae affected N_2_O produced by nitrification and/or denitrification. Organic matter patches were used to create ‘hotspots’ of N_2_O production, a commonly observed phenomenon under natural conditions.

#### Expt 1

**Figure 1:**
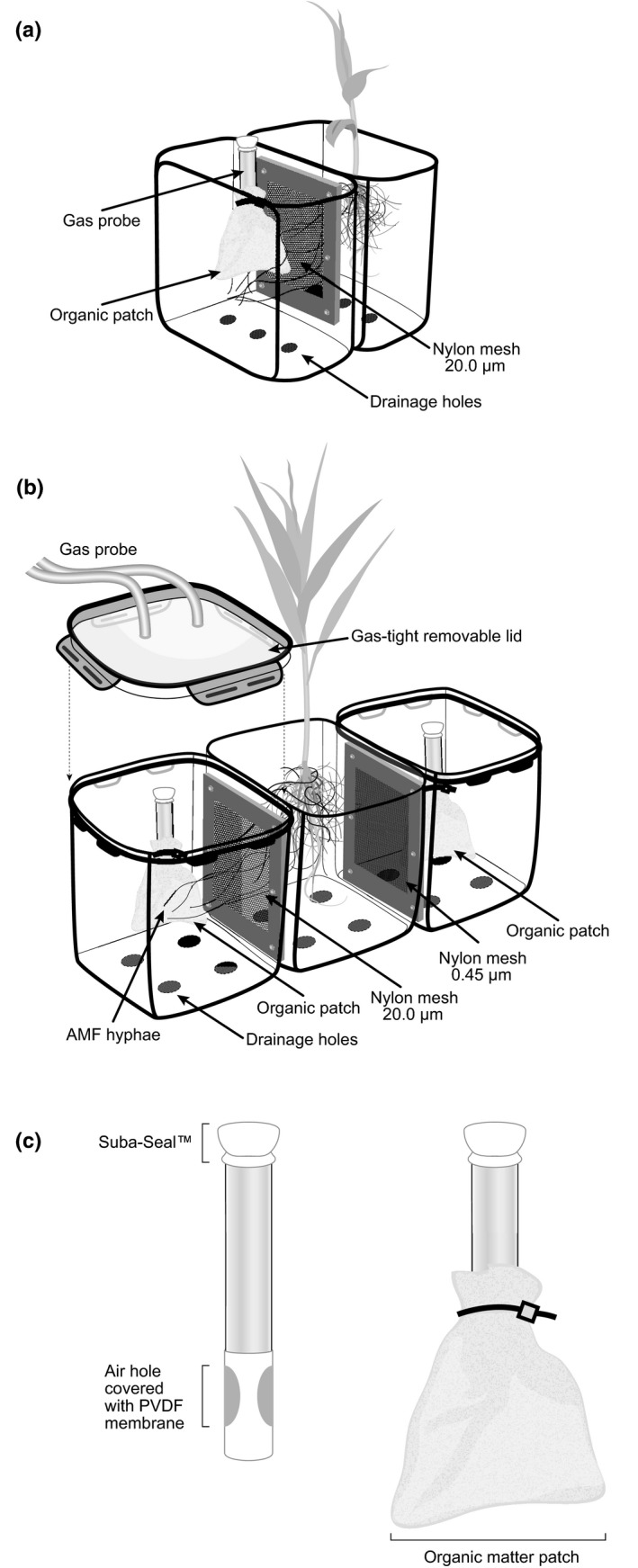
The microcosm units used in Expt 1 (a) and Expt 2 (b) and the organic matter patches and gas probes used in both experiments (c). In Expt 1 the planted compartment was planted with a single *Zea mays* plant and contained the arbuscular mycorrhizal fungal (AMF) inoculum, and the unplanted compartment either allowed or prevented AMF hyphal access. In Expt 2 the central compartment was also planted with a single *Z. mays* plant and contained the AMF inoculum. From the central, planted compartment, the AMF hyphae could access one outer, unplanted compartment (AMF) but not the other (nonAMF). The gas probe was placed within a mesh bag (the ‘organic matter patch’) which contained a mix of dried, milled *Z. mays* leaves and agricultural soil (c). The gas probe and organic matter patch designs were used in both experiments. PVDF, polyvinylidene difluoride.

Microcosm units (Fig. 1a) were constructed by joining two 1 l plastic containers (each 145 × 145 × 70 mm^3^) via a double‐mesh membrane of either 20 μm (John Stanier & Co., Whitefield, Manchester, UK) or 0.45 μm pore size (Osmonics Inc., Minnetonka, MN, USA). These size membranes either allowed (AMF) or denied (nonAMF) AMF hyphal access between the two compartments. In all cases, roots were prevented from passing between the compartments. There were three 6 mm drainage holes in the base of each compartment. In one compartment (the ‘planted’ compartment) a single *Z. mays* seedling (Incredible F1; Mr Fothergills, Newmarket, UK) inoculated with *Rhizophagus irregularis* (PlantWorks Ltd, Kent, UK) was placed, whereas the other compartment contained no plant (the ‘unplanted’ compartment).

#### Expt 2

Three compartment microcosm units were used (Fig. 1b). Each microcosm consisted of a central ‘planted’ compartment (volume, 2 l; dimensions, 150 × 150 × 150 mm; Thumbs Up Ltd, Bury, UK), containing a single *Z. mays* plant inoculated with *R. irregularis*, and on either side of the central planted compartment, two unplanted compartments, separated from the central compartment by a nylon mesh membrane as in Expt 1 (volume, 2.6 l; dimensions, 140 × 140 × 160 mm; Lock & Lock, Australia PTY Ltd, Blacktown, NSW, Australia). The mesh window either allowed AMF hyphal access (AMF; 20 μm mesh) or prevented AMF hyphal access (nonAMF; 0.45 μm mesh) from the central planted to the outer unplanted compartments. A supporting stainless steel mesh (0.25 mm aperture; Mesh Direct, Hanscan Ltd, Burslem, UK) was placed inside the plant compartment over the nylon meshes (0.45 and 20 μm) as a precautionary measure to protect the finer meshes from possible root damage. Thus, each unit had one AMF outer compartment and one nonAMF outer compartment, creating a paired design. The unplanted compartments were covered with a foil layer when the lids were not attached to prevent them from drying out.

#### Expts 1 and 2: growth media

In both experiments, the planted and unplanted compartments contained a mix (1 : 1 v/v) of sand and Agsorb^®^ (Agsorb^®^; Oil‐Dri, Chicago, IL, USA; a calcined attapulgite clay soil conditioner) that had been rinsed thoroughly in deionized water to remove any excess soluble N and/or P. The planted compartments also had 50 g (Expt 1) or 90 g (Expt 2) of a fresh *R. irregularis* inoculum (Plantworks Ltd, Sittingbourne, Kent, UK) and 0.25 g l^−1^ bonemeal (a complex N and P source to encourage mycorrhizal development; 3.5% N, 8.7% P; Vitax, Coalville, Leicestershire, UK). Three pregerminated *Z. mays* seeds were added to each planted compartment for both experiments on 25 June 2012 and thinned to one per pot after 11 d (Expt 1) or 14 d (Expt 2). A sterile centrifuge tube (Expt 1, 15 cm^3^; Expt 2, 50 cm^3^) was added to each of the unplanted compartments to create a hole into which the organic matter patches and gas probes could be added at a later date (see ‘Organic matter patches and gas probes’ section).

### Growth conditions

Microcosm units were placed in a randomized block design in a heated, lit glasshouse. The Experiments ran for 78 d between 25 June and 10 September (Expt 1), and 103 d between 25 June and 5 October 2012 (Expt 2). Photosynthetically active radiation (PAR) was measured weekly for both experiments at plant level in the centre of each block and averaged (± SEM) 141 ± 15 (Expt 1) and 251 ± 45 μmol m^−2^ s^−1^ (Expt 2). Overhead lights were used to extend the photoperiod to 16 h d^−1^ and the mean daily temperatures over the experimental period were 21.9 ± 0.02°C (Expt 1) and 21.5 ± 0.3°C (Expt 2). The planted and unplanted compartments for all microcosm units were watered daily as required. After 2 wk of plant growth, the planted compartments received 50 cm^3^ of a reduced N and P nutrient solution as described by Leigh *et al*. (2009) once a wk (Expts 1 and 2). This was increased to twice weekly at 49 d after planting in Expt 2 and to full N at 55 d after planting as the plants were starting to show symptoms of N deficiency. In Expt 2, at 76 d, the plants began to show P‐deficiency symptoms, so a 3/10 P, full N solution was used once a wk in addition to two 1/10 N and P additions. In total the plants received either 1.74 or 11.97 kg N ha^−1^ in Expts 1 and 2, respectively, over the duration of the experiments (11 and 14 wk, respectively).

### Organic matter patches and gas probes

#### Organic matter patches

Organic matter patch material comprised 13 g DW equivalent agricultural soil (sandy loam; 53°92′N, −1°00′E, pH 6.6 in 0.01 M CaCl_2_ (following Allen, 1974)) mixed with 2 g DW milled *Z. mays* leaves, all enclosed in a 20 μm mesh bag (70 × 60 mm). The mean (± SEM) C and N contents of the mixed organic patches were 1435 ± 182 and 116 ± 15 mg (Expt 1) or 1200 ± 79 and 99  ± 15 mg (Expt 2), respectively, with a C : N ratio of 12 : 1 in both experiments. Each patch contained a gas probe (described in the next section) in the centre (Fig. 1c).

#### Gas probes

A stainless steel tube (9 cm long, outer diameter 1 cm, wall thickness 1 mm; Coopers Needle Works Ltd, Birmingham, UK) was welded at the base to form an air‐tight seal (Fig. 1c). Two diametrically opposed holes, of 6 mm, were drilled through each tube 13 mm from the base. These holes were covered in a polyvinylidene difluoride (PVDF) membrane (0.2 μm; Bio‐Rad) that was air‐permeable but impermeable to water. This fine PVDF membrane was then housed in a supporting silicone tube (wall thickness 0.8 mm, outer diameter 8 mm; Silex Ltd, Lindford, Bordon, Hampshire, UK) with access holes exposing the membrane covering the holes. The stainless steel tube was then sealed at the top with a white rubber Suba‐Seal^®^ (no. 13; Sigma‐Aldrich) to form a gas sampling port. The total internal volume of the gas probe was *c*. 4.5 cm^3^.

A single organic matter patch and gas probe were placed into the preformed holes in the unplanted compartments 2 cm from the mesh window, 7 cm from the surface and covered with sand and Agsorb^®^ media at 29 or 28 d (Expt 1 or 2, respectively) post‐planting.

### Inorganic nitrogen addition

In Expt 1, half of the organic matter patches were injected with 7 cm^3^ of 30 mM NH_4_NO_3_ and the other half with 7 cm^3^ of deionized water (*n *=* *6 in each case) at 44 d after patch addition. Consequently, the treatments were: AMF + NH_4_NO_3_, AMF + water, nonAMF + NH_4_NO_3_ and nonAMF + water. In Expt 2, at 62 d after organic patch addition (90 d after planting) each patch was injected with one of 7 cm^3^ of 15 mM (NH_4_)_2_SO_4_ (NH_4_ treatment), 30 mM KNO_3_ (NO_3_ treatment), 15 mM K_2_SO_4_ (K_2_SO_4_ treatment) or deionized water (water treatment), where the N treatments were equivalent to 0.196 mg N g^−1^ DW patch (*n *=* *10 in each case). In both experiments, two 3.5 cm^3^ aliquots of solution were injected into each organic patch with a 1 h gap between each addition to reduce spread into the surrounding sand/Agsorb^®^.

### Gas sampling and analysis

#### Expt 1

The air in the gas probes was sampled before N addition at 44 d after patch addition. The NH_4_NO_3_ and water addition treatments were then added and the gas probes were sampled again at 24, 48 and 96 h after NH_4_NO_3_ addition. Before sample removal, 1 cm^3^ of N_2_ was added to the probe via the Suba‐Seal, taking care not to disturb the surrounding media. This was left for 10 s before a 1 cm^3^ sample was slowly removed from the gas probe, waiting for a further 5 s to allow the sample to mix inside the syringes before removing the syringe. Each gas sample was then stored in a prefilled 3 cm^3^ Exetainer (Labco Ltd, Lampeter, Ceredigion, UK) (with 6 cm^3^ N_2_), overpressuring the sample to 7 cm^3^ in total. All gas samples were analysed using a gas chromatograph (GC) which quantified the concentration of N_2_O. The concentration (ppm) values for each sample were calculated by comparing with certified standards that were diluted in parallel in a 1 cm^3^ standard: 6 cm^3^ N_2_ ratio and correcting for this dilution. The concentration values were also corrected for dilution from addition of N_2_ to the gas probe just before gas sample removal.

#### Expt 2

Gas sampling was carried out using both gas probes (as described for Expt 1) and continuous flow loop sampling with an attached Los Gatos Isotopic N_2_O analyser (LGR N_2_O; Los Gatos Research Inc., San Jose, CA, USA) which provided an N_2_O concentration once per second. A gas‐tight lid (Fig. 1b) was attached to each of the 80 unplanted compartments in block sequence for a minimum of 5 min, with a minimum of 2 min flushing the system with air between each compartment measurement. Gas sampling using both methods was carried out before N addition (58–59 and 61 d after patch addition), and at 48, 96 and 192 h after N addition (64, 66 and 70 d after patch addition, respectively).

When using the LGR N_2_O analyser, the headspace in the microcosm unit (0.6 l), volume of connecting tubing (0.274 l) and internal volume of the N_2_O analyser (0.850 l) along with the surface area of the soil sampled (0.024 m^−2^) were used in the regression calculation of the N_2_O flux rate in mg m^−2^ h^−1^. These fluxes were calculated using values measured between 200 and 280 s after the cover‐box lid was attached. All regressions were calculated using Sas v.9.3 (SAS institute Inc., Cary, NC, USA).

### Post‐harvest analyses

At harvest, above‐ground material was removed at the soil surface and separated into stalk, flowers, ear, and leaf material. Roots were extracted from the sand/Agsorb^®^ media and washed, and FW and DW of all plant material were recorded. In Expt 1, the dried leaves (green leaves only, defined as > 50% green) were milled and analysed for C and N content using an elemental combustion system (Costech Analytical Technologies Inc., Valencia, CA, USA). The gravimetric water content (g g^−1^ DW) of soil, sand/Agsorb^®^ and patches for each compartment were measured and the AMF extraradical mycorrhizal hyphae (ERM) were extracted from two 5 g (FW) samples from the organic patches and the surrounding growth medium in the unplanted compartments using a modified membrane filter technique (see Staddon *et al*., 1999) and acid fuchsin stain. Hyphal lengths were assessed using the gridline intercept method (Miller & Jastrow, 1992) for a minimum of 50 fields of view at ×125 magnification (using a square grid of 1 cm side length split into 10 × 10 grid sections; Graticules Ltd, Tonbridge, Kent, UK). These hyphal lengths were then converted to ERM length densities (m hyphae g^−1^ soil DW).

### Data analysis

Data were first tested for normality and equality of variance using Kolmogorov–Smirnov and Levene's equality of variance tests, respectively. Statistical analyses were carried out in either Sas v.9.3 or Genstat v.16 (VSN International Ltd, Hemel Hempstead, UK). The pre‐N addition fluxes or concentrations were subtracted from the post‐N addition fluxes or concentrations, respectively, to obtain the change in N_2_O flux or concentration following N addition (referred to ∆N_2_O).

In Expt 1, where N_2_O concentration and ERM length density data did not fulfil normality or equality of variance assumptions, they were log_10_‐transformed. All gas concentration, plant and AMF data were analysed using a two‐way ANOVA, including block, with Duncan's *post hoc* tests. However, transformations on changes in N_2_O concentration following N addition failed to normalize the data, and nonparametric equivalent Friedman's two‐way ANOVAs, including block, with Wilcoxon *post hoc* tests were used. Where N_2_O concentrations were measured over time, repeated‐measures ANOVA, including treatment and block, was used on log_10_‐transformed data. Pearson's product‐moment correlations were used to determine the relationship between variables. Where variables were not normally distributed, Spearman's rank order correlations were used. Untransformed data are presented in all figures.

In Expt 2, differences among treatments were analysed using a two‐way ANOVA including block with Duncan's *post hoc* tests. ERM length density data were log_10_‐transformed before analysis. Where the data failed normality or equality of variance assumptions, nonparametric tests were used. A one‐sample *t*‐test or a Wilcoxon signed‐rank test was used to compare absolute values or differences to zero.

In Expt 2, the ∆N_2_O data were not normally distributed and therefore a Friedman's nonparametric two‐way ANOVA, controlling for block, with Mann–Whitney *U*‐test (unpaired data) or Wilcoxon signed rank (paired data) *post hoc* test and an applied false discovery rate correction were used. Where comparisons in ∆N_2_O flux or ∆N_2_O concentration data were made over time, a nonparametric Friedman's repeated‐measures analysis was used. The relationships between the ∆N_2_O flux and ∆N_2_O concentration for each gas sample following N addition (48, 96 and 192 h post‐N addition) were determined using a Spearman rank order correlation.

There was hyphal breakthrough in one of the nonAMF compartments (treatment: nonAMF, K_2_SO_4_) and therefore this microcosm was excluded from the subsequent data analyses. In addition, the N_2_O concentration for one experimental unit in the AMF treatment (treatment: (NH_4_)_2_SO_4_) was out of range on the GC for the sample taken 48 h post‐N addition and therefore these AMF and nonAMF N_2_O concentration values were also omitted.

## Results

In the AMF treatments, *R. irregularis* colonized the organic matter patches successfully in both experiments, with ERM length densities of 1.23 ± 0.25 m g^−1^ DW in Expt 1 (nonAMF, 0.31 ± 0.05 m g^−1^ DW; *F*_1,12_ = 30.77, *P *=* *0.0001) and 0.88 ± 0.08 m g^−1^ in Expt 2 (nonAMF, 0.35 ± 0.04 m g^−1^ DW; *t*_39_ = 8.993, *P *<* *0.0001).

### Pre‐N addition N_2_O production

**Figure 2:**
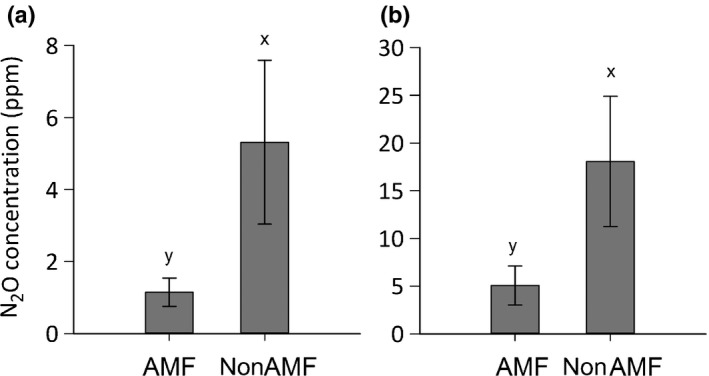
Mean N_2_O concentration (ppm) in arbuscular mycorrhizal fungal access (AMF) and no AMF access (nonAMF) organic matter patches at 43 d after patch addition in Expt 1 (a) and at 58 d after patch addition in Expt 2 (b). Error bars are ± SEM (a, *n *=* *12; b, *n *=* *39). Different letters represent significant differences at *P *<* *0.05 as determined using: (a) two‐way ANOVAs; and (b) by comparing the ∆AMF value with zero (Wilcoxon signed‐rank test).

Before inorganic N addition there was a greater concentration of N_2_O in the nonAMF patches than in the AMF patches in both experiments (Fig. 2; Expt 1, *F*_1,12_ = 6.46, *P *=* *0.026; Expt 2, *S*_38_ = −186, *P *=* *0.0076). A similar trend (at the *P *<* *0.1 level) was found for the N_2_O fluxes in Expt 2, with greater N_2_O fluxes measured from the nonAMF compartments than from the AMF compartments (*S*_38_ = −128, *P *=* *0.074). In Expt 2, N_2_O fluxes measured by continuous flow loop sampling were positively correlated with the patch N_2_O concentrations measured using gas probes (*r*_s_ = 0.7495, *P *<* *0.0001). As N_2_O production is inherently variable, this degree of consistency both between and within experiments is striking, particularly because it was observed in the absence of any additional applied inorganic N. In both experiments, there was no significant correlation between the pre‐N addition AMF treatment N_2_O concentration or fluxes and the ERM lengths (*P *>* *0.05 in each case).

### Post‐N addition and harvest

#### Expt 1

**Figure 3:**
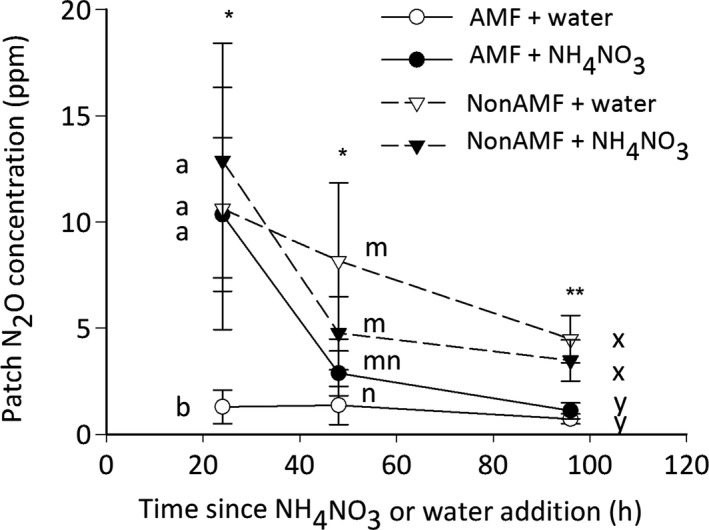
Mean patch nitrous oxide (N_2_O) concentration at 24, 48 and 96 h after addition of inorganic nitrogen (N) (NH_4_ NO_3_: closed symbols) or water (open symbols) for arbuscular mycorrhizal fungal access patches (AMF; solid lines) and no AMF access patches (nonAMF; dashed lines) shown over time. Error bars are ± SEM (*n *=* *6). Asterisks represent a significant difference among treatments within each sample period (*, *P *<* *0.05; **, *P *<* *0.01) as determined using a two‐way ANOVA. Different letters within each sample timing represent significant differences between treatments for that sample timing (*P *<* *0.05).

In Expt 1 the highest patch N_2_O concentrations were observed 24 h after the application of inorganic N or water in all treatments except AMF + water, demonstrating the rapid response of N_2_O producers to treatment application. The patch N_2_O concentrations of the nonAMF and AMF + NH_4_NO_3_ treatments subsequently decreased over time. By contrast, the AMF + water patch N_2_O concentration remained low. Consequently, there was a significant effect of both time and treatment on patch N_2_O concentration in addition to a significant interaction between these two factors (Fig. 3; time, *F*_2,30_ = 4.37, *P *=* *0.023; treatment, *F*_3,15_ = 5.67, *P *=* *0.0084; time × treatment, *F*_6,30_ = 3.23, *P *=* *0.015). These results therefore demonstrate how rapidly N_2_O production rates can change over time and emphasize the requirement for repeated measurements following inorganic N application. Two‐way ANOVAs at each time point showed that the N_2_O concentration of the AMF + water treatment was lower than all other treatments at 24 h post‐treatment application (Fig. 3; *F*_3,15_ = 4.44, *P *=* *0.020). This effect decreased by the 48 h sample, although the nonAMF + water and nonAMF + NH_4_NO_3_ treatments still had a higher N_2_O concentration than that of the AMF + water treatment (*F*_3,15_ = 4.95, *P *=* *0.014). At 96 h post‐treatment application, the AMF patch N_2_O concentrations were not significantly different from each other but were significantly lower than those of the nonAMF patches (*F*_3,15_ = 7.25, *P *=* *0.0031). At 24 h post‐treatment application, the ∆N_2_O concentration was higher in both the AMF + NH_4_NO_3_ and nonAMF + NH_4_NO_3_ treatments than in the AMF + water treatment (*Q*_3_ = 8.2, *P *=* *0.042). However, the nonAMF + water treatment was not significantly different from the AMF + NH_4_NO_3_ treatment or nonAMF + NH_4_NO_3_ treatment.

There was no relationship between the AMF ERM length densities and N_2_O concentration in the AMF patches at any point (*P *>* *0.05 in each case) and the moisture contents of the organic patches did not differ among treatments at harvest (Q_3_ = 0.707, *P *=* *0.871). Additionally, there was no significant difference (*P *>* *0.05) in total plant DW or the DW of the various plant tissues (i.e. leaf, total shoot, stalk, total root, root weight ratio, tassel) between the AMF and nonAMF treatments (see Supporting Information Table S1). The addition of NH_4_NO_3_ or water had no effect on the leaf C and N content or concentrations or on the C : N ratios (*P *>* *0.05 in each case), and therefore these data were combined for comparison of the AMF with the nonAMF treatments. Leaf C content did not differ between AMF and nonAMF plants (Table 1; *F*_1,12_ = 0.30, *P *=* *0.595), although the leaf C concentrations were lower in the AMF than in the nonAMF treatments (Table 1; *F*_1,12_ = 5.37, *P *=* *0.039). Both the N content (Table 1; *F*_1,12_ = 14,18, *P *=* *0.0023) and concentration (*F*_1,12_ = 20.06, *P *=* *0. 0008) of the leaves were higher in the AMF than in the nonAMF treatments. Consequently, the C : N ratio of the leaves was lower in the AMF than in the nonAMF treatments (Table 1; *F*_1,12_ = 18.51, *P *=* *0.001). However, the organic patch N_2_O concentration was not significantly related to the leaf C or N content or concentration, or to the leaf C : N ratio, either before or after N addition, for both the AMF and nonAMF treatments (*P *>* *0.05 in each case).

**Table 1:** Mean (± SEM) leaf nitrogen (N) and carbon (C) total content and concentration, and C : N ratio of *Zea mays* leaves from arbuscular mycorrhizal fungi (AMF) and nonAMF treatments in Expt 1  (*n *=* *12)

		AMF	NonAMF
Leaf N	Total content (mg)	**13.8 ± 0.8 a**	**10.2 ± 0.9 b**
	Concentration (mg g^−1^ DW)	**11.3 ± 0.6 f**	**8.8 ± 0.5 g**
Leaf C	Total content (mg)	503.2 ± 19.9 j	488.1 ± 27.2 j
	Concentration (mg g^−1^ DW)	**413.4 ± 2.8 m**	**422.9 ± 3.7 n**
	Leaf C : N ratio	**37.6 ± 2.0 x**	**50.0 ± 3.0 y**

Different letters within rows represent significant differences at *P *=* *0.05 (in bold) as determined using two‐way ANOVAs.

#### Expt 2

**Figure 4:**
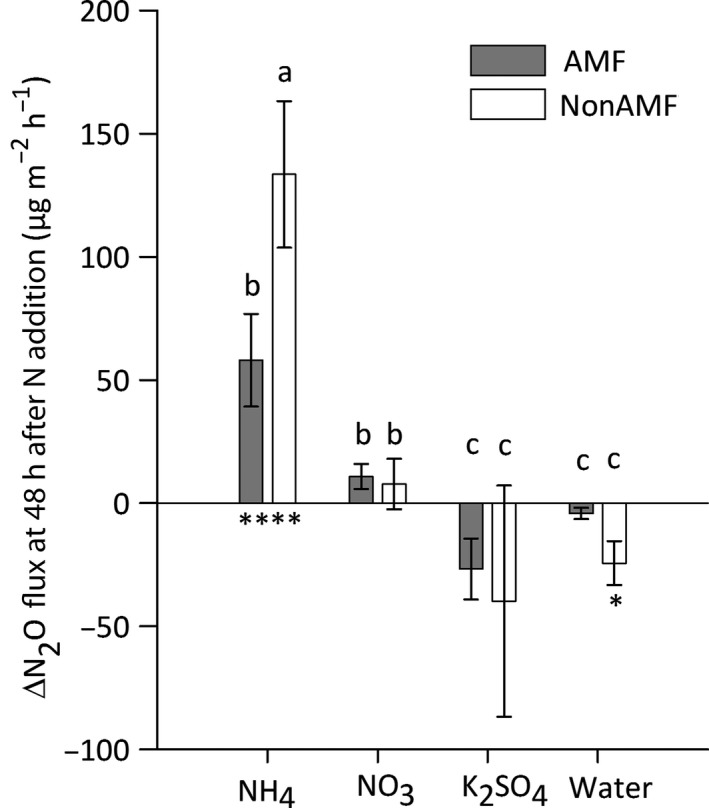
Mean difference between 48 h post‐nitrogen (N) addition (64 d after patch addition) and pre‐N addition (61 d after patch addition) nitrous oxide (N_2_O) fluxes (∆N_2_O flux) for arbuscular mycorrhizal fungal access (AMF; closed bars) and no AMF access (nonAMF; open bars) treatments, split by N‐addition treatment. The N‐addition treatments were (NH_4_)_2_ SO_4_ (labelled as NH_4_), KNO_3_ (labelled as NO_3_), K_2_ SO_4_ or water. Bars with different letters are significant at *P *=* *0.0018 as determined by Mann–Whitney *U* or Wilcoxon signed‐rank *post hoc* tests with a false discovery rate correction applied. Asterisks below the bars indicate significant differences from zero (*, *P *<* *0.05; **, *P *<* *0.01). Error bars are ± SEM (*n *=* *10).

There was a significant difference in ∆N_2_O fluxes between the inorganic N and water application treatments at 48 h post‐application (Fig. 4; *Q*_7_ = 44.85, *P *<* *0.0001). In both the AMF and nonAMF patches, more N_2_O was produced following addition of NH_4_^+^ than for any other treatment. Strikingly, however, *c*. 2.5 times more N_2_O was produced from the nonAMF than from the AMF treatment (Fig. 4; *S*_9_ = −26.5, *P *=* *0.0084). These differences then declined by the 96 h sample and were no longer significant at the 192 h sample, again illustrating the transient nature of N_2_O release and the importance of following the fluxes over discrete timescales (Table 2). There was no significant difference in the % moisture content of either the patch or sand/Agsorb^®^ medium between the AMF and nonAMF treatments at destructive harvest (patch, *t*_39_ = −0.26, *P *=* *0.799; sand/Agsorb^®^, *S*_39_ = −47, *P *=* *0.519).

**Table 2:** Expt 2: Friedman's test statistics controlling for block comparing the post‐nitrogen (N) minus pre‐N (61 d post‐patch addition) patch nitrous oxide (N_2_O) concentrations (∆N_2_O concentrations) or compartment N_2_O fluxes (∆N_2_O fluxes) among N‐addition treatments, for each of the gas sampling events

	Time since N addition								
	48 h			96 h			192 h		
	*Q*	d.f.	*P*	*Q*	d.f.	*P*	*Q*	d.f.	*P*
Patch ∆N_2_O concentration	28.89	7	**0.0002** ***	14.35	7	**0.045** *	3.79	7	0.804
Compartment ∆N_2_O flux	44.85	7	**< 0.0001** ***	25.63	7	**0.0006** ***	4.80	7	0.684

*Q*, Friedman's test statistic; d.f., degrees of freedom; *n *=* *10. Significant results are indicated in bold at *P *=* *0.05 (*, *P *<* *0.05; ***, *P *<* *0.001).

## Discussion

This is the first study to show that N_2_O production is reduced as a direct consequence of the presence of AMF hyphae. Moreover, this reduction was demonstrated in both the presence and, notably, the absence of applied inorganic N, indicating that this is a persistent effect. Studies to date have indicated that AMF may influence soil N_2_O production, but this has always been in the presence of plant roots and additional inorganic N (Lazcano *et al*., 2014; Bender *et al*., 2015). Critically, the finding that N_2_O production was reduced when AMF hyphae, but not plant roots, were present was consistent between the two independent experiments reported here.

Previous studies have applied inorganic N and assessed the N_2_O flux from the mycorrhizosphere (i.e. the soil influenced by AM colonized roots and AMF hyphae), often only at a single time point after N application, potentially masking cumulative effects (Bender *et al*., 2015). AMF hyphae can extend far beyond the plant roots alone, with the ERM being 10 times larger, in biomass terms, than the intraradical mycelium (Olsson *et al*., 1999). Thus, the influence of AMF hyphae on soils (in the ‘hyphosphere’) will extend beyond the zone of influence of roots alone, and studies to date have not explored this widespread zone of hyphal influence on N_2_O production in isolation. Furthermore, as the mycorrhizosphere includes both AMF‐colonized plant roots and AMF hyphae, it is impossible to know whether any effect is a consequence of the AMF hyphae or roots, or both. Rhizodeposition differs between AM and nonAM plants (Jones *et al*., 2004), while C exudation from AMF hyphae may also result in quantitative and qualitative changes in the total C flux into the soil (Toljander *et al*., 2007). Moreover, AMF hyphae influence N cycling through the capture of N and subsequent transfer of at least some of this N to their associated host plant (Leigh *et al*., 2009; Thirkell *et al*., 2016). C and N are key controls of denitrification and nitrification rates (Bollmann & Conrad, 1998; Hino *et al*., 2010). It is not possible, therefore, to separate AMF and root control of N_2_O fluxes in the mycorrhizosphere without first separating the AMF hyphae from the plant roots.

Nevertheless, there is some evidence of AMF interacting with soil N_2_O production in the mycorrhizosphere, although results have been inconsistent. Bender *et al*. (2015) found that the N_2_O flux was lower following the application of NO_3_^−^ in the AM mycorrhizosphere when compared with the rhizosphere of a nonAM control. By contrast, Cavagnaro *et al*. (2012) found no effect of AM plants on N_2_O production, whereas Lazcano *et al*. (2014) found a reduction in N_2_O in the mycorrhizosphere of AM plants. Thus, there is support for AMF resulting in reduced N_2_O production in the mycorrhizosphere, but the cause of this reduction has so far been poorly understood, probably because of confounding effects of the host plant root system also being present. Hypotheses for the decreased N_2_O production in the mycorrhizosphere included a reduction in denitrification (Bender *et al*., 2015) and increased water use by AM plants (Lazcano *et al*., 2014).

In this study, the finding of reduced N_2_O production in the presence of AMF hyphae was evident even before inorganic N application. There was also evidence for an increase in both leaf N content and concentration when the AMF had access to the organic matter patches. This suggests that the AMF were supplying their host plant with additional N, presumably from the organic matter patch, as all planted compartments received the same quantity of nutrient solution. Whilst there is a wide range in reported contribution of AMF to plant N (reviewed by Hodge & Storer, 2015), the findings in this study are in agreement with previous investigations using ^15^N that substantial quantities of N can be transferred from the patch to the plant via AMF hyphae (Leigh *et al*., 2009; Thirkell *et al*., 2016).

The inorganic N applications here were used as a tool to identify the pathway of N_2_O production being influenced by the AMF hyphae. The addition of NO_3_^−^ did not result in increased N_2_O production from any treatment, suggesting that in this study, denitrification was not a key factor in controlling N_2_O production. There was also no significant difference in gravimetric water content of the organic matter patches, or the surrounding sand/Agsorb^®^ medium at harvest. Thus, these factors were not important controls of N_2_O production in the present study. Instead, we found direct evidence for a reduction in N_2_O produced via nitrification in the presence of AMF hyphae. This is a critical finding and may help to explain variable N_2_O fluxes under field conditions. As agricultural soils are one of the largest sources of N_2_O, it is highly relevant that the soil used here was agricultural in origin, and the plant material for the organic matter patches was *Z. mays*, a globally important crop (Leff *et al*., 2004).

**Figure 5:**
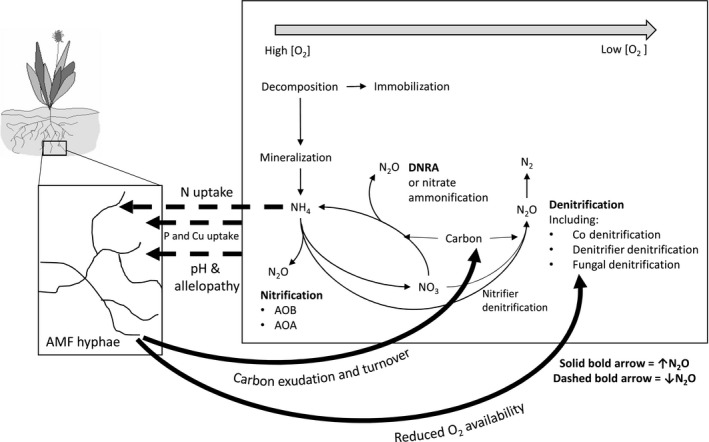
Summarized potential interactions between arbuscular mycorrhizal fungal (AMF) hyphae and soil nitrous oxide (N_2_O)‐producing processes as described in Baggs (2011) and Zhu *et al*. (2013). The solid and dashed bold lines represent AMF effects that could result in an increase and decrease in N_2_O production, respectively. AMF can affect the availability of nitrogen (N), phosphorus (P), copper (Cu) and iron (Fe) in soils, as well as potentially changing soil pH. Nitrifier nitrification is generally carried out by ammonia‐oxidizing bacteria (AOB) and archaea (AOA). Dissimilatory reduction of nitrate to ammonium (DNRA) may produce N_2_O as a side product. DNRA is also known as nitrate ammonification. There are various pathways and organisms capable of carrying out these roles, but, for simplicity, they are grouped by factors affecting the rate of N_2_O production (i.e. availability of O_2_, or carbon).

The soil N_2_O fluxes in this study were predominantly controlled by the availability of NH_4_^+^. These fluxes were monitored at intervals up to 192 h after inorganic N application, by which point the N_2_O peak declined back to pre‐N application values, thus ensuring that the full response period was recorded. There was a significantly greater N_2_O flux in response to NH_4_^+^ addition in the nonAMF than in the AMF treatment, indicating reduced N_2_O production via nitrification in the presence of AMF hyphae. The current understanding of the main pathways of N_2_O production in soils (as described in Baggs, 2011; Zhu *et al*., 2013) are shown in Fig. 5 together with the potential mechanisms by which AMF may interact with N_2_O production. If NH_4_^+^ elicits N_2_O production but NO_3_^−^ application does not, by process of elimination the pathway involved in N_2_O production must be a nitrification pathway.

The links between AMF presence and reduced nitrification rates are in broad agreement with a series of one field‐based and three mesocosm‐based studies by Veresoglou *et al*. (2011). The potential nitrification rates were lower in the mycorrhizospheres of AM plants than in those from weakly AM mycorrhizospheres (Veresoglou *et al*., 2011). The *nirK* gene, responsible for N_2_O production has also been shown to be negatively correlated with AMF abundance (Bender *et al*., 2014). Thus, the presence of AM plants may reduce N_2_O production by reducing nitrification rates. Our present study demonstrates, for the first time, that AMF hyphae have a direct and limiting influence on soil N_2_O produced via nitrification, independent of any plant root influence.

The main ‘nitrification’ pathways in soil potentially resulting in N_2_O release are nitrifier nitrification, and nitrifier denitrification. Nitrifier nitrification is an aerobic process and can be carried out by ammonia‐oxidizing bacteria (AOB), archaea (AOA), and organisms capable of complete ammonia oxidation (comammox) (Daims *et al*., 2015; van Kessel *et al*., 2015). AOB and AOA have also been shown to produce N_2_O (Jiang & Bakken, 1999; Jung *et al*., 2014). Nitrifier denitrification is also carried out by autotrophic nitrifiers, and can be a significant source of N_2_O (Wrage *et al*., 2001; Kool *et al*., 2011b). Thus, there are various pathways by which the N_2_O in this study may have been produced following the application of NH_4_^+^ and consequently reduced by the presence of AMF hyphae (Fig. 5).

Regardless of the process, the response to NH_4_^+^ application in the AMF treatments suggests that there was either a reduction in N_2_O production, through reduced function or number of nitrifiers, or that nitrifier activity was masked by an increase in activity of N_2_O reducers, which can cause some soils to become N_2_O sinks (Domeignoz‐Horta *et al*., 2017). It is also feasible that the presence of AMF hyphae modified the microbial community, shifting it away from N_2_O‐producing nitrifiers or nitrifier denitrifiers, perhaps towards organisms capable of complete nitrification (van Kessel *et al*., 2015) or N_2_O reduction (Sanford *et al*., 2012; Jones *et al*., 2014; Domeignoz‐Horta *et al*., 2017).

Domeignoz‐Horta *et al*. (2017) found that N_2_O hotspots were predominantly controlled by changes in the microbial communities, whereas lower N_2_O‐producing areas were more likely to be controlled by variation in soil properties. Using similar organic patches as in the present study, Nuccio *et al*. (2013) found that while there was no overall change in bacterial diversity, the presence of AMF hyphae significantly modified the bacterial community. Interestingly, Gemmatimonadetes and Deltaproteobacteria were two of four bacterial phylum that had a higher relative abundance in response to the presence of AMF hyphae in the litter (Nuccio *et al*., 2013). Both the Gemmatimonadetes and Deltaproteobacteria have subsequently been found to posses *nosZ* genes, and can thus utilize exogenous N_2_O as an electron acceptor (Jones *et al*., 2013; Park *et al*., 2017). AMF abundance has also been found to positively correlate with *nosZ* gene abundance (Bender *et al*., 2014). This, together with the large export of N from the patch by the AMF hyphae and the resulting modifications in the physicochemical environment in the decomposing litter patch, may contribute to a reduction in N_2_O emissions.

Given the evidence that AMF are known to have a high N demand (Hodge & Fitter, 2010), one hypothesis could be that AMF hyphae were eliciting a longer‐term control on the nitrifying community, as nitrifiers are inherently slow‐growing, taking from 8 h up to a number of days to double in number (Belser & Schmidt, 1980; Woldendorp & Laanbroek, 1989; Prosser, 2007; Prosser & Nicol, 2012). AMF hyphae are thought predominantly to take up inorganic N in the form of NH_4_^+^ (Govindarajulu *et al*., 2005; Tanaka & Yano, 2005), and AOB are generally thought to be poor competitors for NH_4_^+^ (Verhagen *et al*., 1995; Bollmann *et al*., 2002). The AMF hyphae may therefore have reduced the amount of available NH_4_^+^ in the hyphosphere, resulting in a reduction in the population of active AOB. If AOB were the main N_2_O producers, this may explain the reduced N_2_O production before inorganic N application when the AMF hyphae were present. It may also explain the lack of N_2_O production in the presence of AMF hyphae when NH_4_^+^ was applied, i.e. the AOB population may have been small and too slow‐growing to respond to the inorganic NH_4_^+^ supplied, which may have, instead, been taken up by the N‐rich AMF hyphae.

While AMF may increase or decrease the pH of surrounding media, thought to be a consequence of NO_3_^−^ or NH_4_^+^ uptake, respectively (Li *et al*., 1991; Bago *et al*., 1996), the relative importance of pH effects on N_2_O production if C, NH_4_^+^ or NO_3_^−^ are limiting is not clear (reviewed by Šimek & Cooper, 2002), with both increased and decreased nitrification‐derived N_2_O production reported under low‐pH conditions (Mørkved *et al*., 2007; Cheng *et al*., 2013). The patch pH was not measured in this study, and potential changes in pH cannot be fully discounted. However, the implications of N, and more importantly the form of N, exported by AMF on the local physicochemical properties, including pH, warrant further attention. This may also aid in explaining the differing impacts reported for AMF on decomposition processes, and their importance not only for N, but also for C cycling and stabilization processes (Hodge, 2001; Hodge *et al*., 2001; Cheng *et al*., 2012).

In order to fully understand the mechanism for the reduction in N_2_O production via nitrification observed in the presence of AMF hyphae found in this study, further research should focus upon gene expression and the responses of the microbial community, including nitrifier communities, AOA, AOB and potential nondenitrifying N_2_O reducers. Monitoring would also help to establish if nitrifier populations were suppressed by the presence of AMF hyphae, as we suggest. Furthermore, field‐based studies using a wider range of soil types and environmental conditions are an essential next step to determine the global scale and significance of this interaction in both natural and agricultural systems.

In conclusion, using two independent glasshouse‐based experiments, we have found that the presence of AMF hyphae reduced the production of the globally important greenhouse gas, N_2_O. Cropped agricultural soils cover a significant proportion of land area, representing 28.4% of agricultural land, or 10.9% of the total global land area in 2011 (FAO, 2017). The diversity of AMF is reduced in agricultural soils (Helgason *et al*., 1998), and these soils are one of the largest contributors to N_2_O emissions. This study suggests that a reduction in the presence of AMF may contribute to further increases in N_2_O production. This could have significant implications for better management of agricultural soils in the future. Given the ubiquity of the AM association, including under agricultural situtations, these findings have global implications not only for our fundamental understanding of the mechanisms of soil N cycling, but also for greenhouse gas management and climate change mitigation.

## Author contributions

K.S., P.I. and A.H. designed the research; K.S. performed the research and conducted all data analysis; A.C. performed practical work for Expt 1; and K.S. and A.H. wrote the manuscript.

## Data accessibility

Data created during this research are available by request from the University of York Data Catalogue. https://doi.org/10.15124/67decab3-9ea6-4cde-812e-3c762eba2ec6.

## Supporting information

Please note: Wiley Blackwell are not responsible for the content or functionality of any Supporting Information supplied by the authors. Any queries (other than missing material) should be directed to the *New Phytologist* Central Office.

**Table S1** Mean plant biomass parameters from AMF and nonAMF treatments in Expt 1
