# Supplementary material for: Arbuscular mycorrhizal fungi reduce nitrous oxide emissions from N2O hotspots
Source: New Phytol. 2017 Dec 5;220(4):1285–95. doi: 10.1111/nph.14931 (PMC6282961; doi:10.1111/nph.14931)
Supplement: Supplementary file 1 — Table S1 Mean plant biomass parameters from AMF and nonAMF treatments in Expt 1 [file NPH-220-1285-s001.pdf]

New Phytologist **Supporting Information**

Article title: **Arbuscular mycorrhizal fungi reduce nitrous oxide emissions from N_2O hotspots**

Authors: Kate Storer, Aisha Coggan, Phil Ineson, Angela Hodge

Article acceptance date: 26th October 2017.

The following Supporting Information is available for this article:

Table S1. Mean plant biomass parameters from AMF and non-AMF treatments in Experiment 1 ± standard error of the mean (n = 12). There were no significant differences between treatments for any of the parameters ($P > 0.05$ in each case) as determined using two-way ANOVAs.

	AMF	Non-AMF
Leaf DW (g)	1.22[a] ± 0.05	1.16[a] ± 0.07
Stalk DW (g)	2.08[b] ± 0.08	1.97[b] ± 0.11
Tassel DW (g)	0.03[c] ± 0.01	0.02[c] ± 0.003
Total shoot DW (g)	3.89[d] ± 0.32	3.82[d] ± 0.33
Root DW (g)	1.24[e] ± 0.29	1.68[e] ± 0.45
Total plant DW (g)	5.13[f] ± 0.41	5.50[f] ± 0.57
Root weight ratio	0.23[g] ± 0.03	0.27[g] ± 0.05

Mean values with the same letters are not significantly different at $P = 0.05$.
